# Hyperoxia Reversibly Alters Oxygen Consumption and Metabolism

**DOI:** 10.1100/2012/410321

**Published:** 2012-05-01

**Authors:** Patrick Lauscher, Sabine Lauscher, Harry Kertscho, Oliver Habler, Jens Meier

**Affiliations:** ^1^Department of Anesthesiology and Intensive Care Medicine, Tübingen University Hospital, Eberhard-Karls University, 72076 Tübingen, Germany; ^2^Clinic of Anesthesiology, Intensive Care Medicine, and Pain Control, Nord-West Hospital, 60488 Frankfurt, Germany; ^3^Clinic of Anesthesiology, Intensive Care Medicine and Pain, Therapy Goethe-University Hospital Center, 60590 Frankfurt, Germany

## Abstract

*Aim*. Ventilation with pure oxygen (hyperoxic ventilation: HV) is thought to decrease whole body oxygen consumption (VO_2_). However, the validity and impact of this phenomenon remain ambiguous; until now, under hyperoxic conditions, VO_2_ has only been determined by the reverse Fick principle, a method with inherent methodological problems. The goal of this study was to determine changes of VO_2_, carbon dioxide production (VCO_2_), and the respiratory quotient (RQ) during normoxic and hyperoxic ventilation, using a metabolic monitor. *Methods*. After providing signed informed consent and institutional acceptance, 14 healthy volunteers were asked to sequentially breathe room air, pure oxygen, and room air again. VO_2_, VCO_2_, RQ, and energy expenditure (EE) were determined by indirect calorimetry using a modified metabolic monitor during HV. *Results*. HV reduced VO_2_ from 3.4 (3.0/4.0) mL/kg/min to 2.8 (2.5/3.6) mL/kg/min (*P* < 0.05), whereas VCO_2_ remained constant (3.0 [2.6/3.6] mL/kg/min versus 3.0 [2.6/3.5] mL/kg/min, n.s.). After onset of HV, RQ increased from 0.9 (0.8/0.9) to 1.1 (1.0/1.1). Most changes during HV were immediately reversed during subsequent normoxic ventilation. *Conclusion*. HV not only reduces VO_2_, but also increases the respiratory quotient. This might be interpreted as an indicator of the substantial metabolic changes induced by HV. However, the impact of this phenomenon requires further study.

## 1. Introduction

Oxygen (O_2_) is widely used in emergency medicine as an acute measure for many different pathologies. Most of the emergency guidelines, such as for acute myocardial infarction or hemorrhagic shock, include usage of supplemental oxygen with the aim to improve macrohemodynamics, oxygen transport, and tissue oxygenation [1–4]. However, the application of pure oxygen is associated with side effects, including hyperoxic arteriolar constriction and reduced functional capillary density, which reduces nutritive organ blood flow and increases peripheral oxygen shunting [5–8]. 

Despite these negative side effects, hyperoxic ventilation is thought to prevent tissue hypoxia by other means: Chapler et al. were among the first to recognize that breathing 100% O_2_ significantly decreases oxygen consumption and optimizes oxygen delivery—oxygen consumption balance [9], a phenomenon that has been confirmed [10–14]. However, it is not known whether this repeatedly observed VO_2_ decrease after onset of hyperoxic ventilation is not merely the result of erroneous measurement, since all data collected thus far have been obtained by the reverse Fick method from data obtained by a pulmonary artery catheter (cardiac output [CO], arterial oxygen content [CaO_2_], and venous oxygen content [CvO_2_]). There are several methodological weaknesses inherent to this indirect calculation of VO_2_ that make results interpretation difficult [15–17].

Of note, however, VO_2_ can not only be calculated but also directly measured using a metabolic monitor for low inspiratory oxygen fractions (FiO_2 _< 0.6). Although theoretically possible, VO_2_ measurement up to an inspiratory oxygen fraction of 100% has not been implemented to a metabolic monitor so far. As a consequence, no study exists, where VO_2_ has actually been directly measured during HV. In contrast to the Fick method, a metabolic monitor makes it possible to measure concomitant changes of carbon dioxide production (VCO_2_) and the respiratory quotient (RQ) during HV. Changes in these 2 important indicators of oxygen balance may facilitate interpretation of the observed changes in VO_2_.

The aim of this study was to determine VO_2_, VCO_2_, and RQ during normoxic and hyperoxic ventilation in healthy volunteers by means of a modified metabolic monitor, especially designed for VO_2_ measurement during HV (Oxycon Pro, VIASYS Healthcare, Hoechberg, Germany). We hypothesized that HV not only decreases VO_2_ but also alters VCO_2_ and RQ, probably indicating substantial changes in oxygen metabolism during HV versus normoxic ventilation.

## 2. Materials and Methods

### 2.1. Study Design

Following approval by the local ethics committee and informed consent, the experiments were performed in 14 volunteers (7 men and 7 women) as a single blinded, nonrandomized cross-over study.

### 2.2. Measurement of VO_2_ and VCO_2_

Volunteers were connected to a modified metabolic monitor (Oxycon Pro, VIASYS Healthcare, Hoechberg, Germany) that is designed to measure VO_2_, VCO_2_, and RQ during hyperoxic ventilation. The basic version of this metabolic monitor has been thoroughly described and validated elsewhere [18]. Experimental measurements of VO_2_ and VCO_2_ were obtained by calibrating the metabolic monitor with the inspiratory oxygen concentration of every time point (room air, pure oxygen, and room air) and applying a modified, validated Haldane equation. Expired gas was passed through a flow meter, oxygen analyzer, and carbon dioxide analyzer. The flow meter and gas analyzers were connected to a computer, which calculated minute ventilation, oxygen uptake (VO_2_), carbon dioxide production (VCO_2_), the respiratory quotient (RQ), and energy expenditure (EE) each minute, from adapted equations for hyperoxic ventilation. Values obtained over 20 min were averaged and are given as the median value for each time point.

#### 2.2.1. Participants

Fourteen healthy nonsmoking volunteers (7 men and 7 women) agreed to participate in this study. Health histories and physical examinations were completed, and written informed consent was obtained according to protocols approved by the University of Frankfurt ethics committee. Prior to the experiments, the subjects were interviewed and examined for the following exclusion criteria: neurological, cardiovascular, pulmonary, hepatic, renal, hematopoietic, gastrointestinal, metabolic, or psychiatric dysfunction; receiving medication on a regular basis. Subjects' physical characteristics were as follows: age 29.3 (range: 24–37) yrs; height 176 cm (range: 162–198 cm); weight 74.5 kg (range: 53–105 kg).

### 2.3. Experimental Protocol

Measurements were made as subjects watched television while seated in a beach-chair position in a temperature-controlled room (21°C). Measurements were made using the metabolic monitor connected to an intensive care respirator (Vela, VIASYS Healthcare, Hoechberg, Germany). The gas mixture was administered through a nonrebreathing system with a tightly fitted facemask. The resistance of the breathing system was not compensated for by pressure support throughout the protocol. No continuous positive airway pressure was applied, since volunteers had no artificial airway. The inspiratory oxygen fraction was controlled by oxygen sensors in the circuit. After 30 min of adaptation, the volunteers sequentially breathed room air (FiO_2_ 0.21; time point *NOX 1*), pure oxygen (FiO_2_ 1.0; time point *HOX*), and room air (FiO_2_ 0.21; time point *NOX 2*) again for 20 min each. Before each measurement, the metabolic monitor was recalibrated according to the manufacturer's instructions. After each change in FiO_2_, an equilibration period of 8 min was allowed to elapse, to achieve steady state conditions. We demonstrated in 3 pilot experiments that after a wash-in phase of 5 min, a steady-state oxygen uptake is reached, and any changes in VO_2_ cannot be attributed to wash-in kinetics after this time period. All the volunteers were blinded to the FiO_2_ used; however, the different FiO_2_ were not applied in a randomized order.

### 2.4. Monitoring

Brachial blood pressure was recorded at 5 min intervals by a semiautomated noninvasive oscillometric sphygmomanometer (Datascope Passport, NJ, USA). Pulse oximeter saturation (SpO_2_) was monitored noninvasively by a standard anesthesia monitor (Datascope Passport, NJ, USA). A digital 12-channel ECG recording was registered continuously throughout the protocol (Cardiax Mesa, Benediktbeuren, Germany). VO_2_, VCO_2_, RQ, and EE were determined as described above. No further invasive measurements have been established.

### 2.5. Statistical Analysis

Data are presented as medians (Q1-Q3). Calculations and statistical analysis were performed with the R software package (R-Project, 2.2.0, R-Foundation, Vienna, Austria). Distribution of data was tested by a Shapiro-Wilks test. Because not all data were normally distributed, differences between *NOX 1, HOX, *and *NOX 2* were analyzed with a Wilcoxon-signed rank test. Post hoc analysis of differences detected with the Wilcoxon signed-rank test was performed by the Bonferroni-Holm method. Overall, statistical significance was accepted at *P* < 0.05.

## 3. Results

All the 14 volunteers completed the study, and none reported discomfort from the facemask or the administration of pure oxygen.

**Figure 1:**
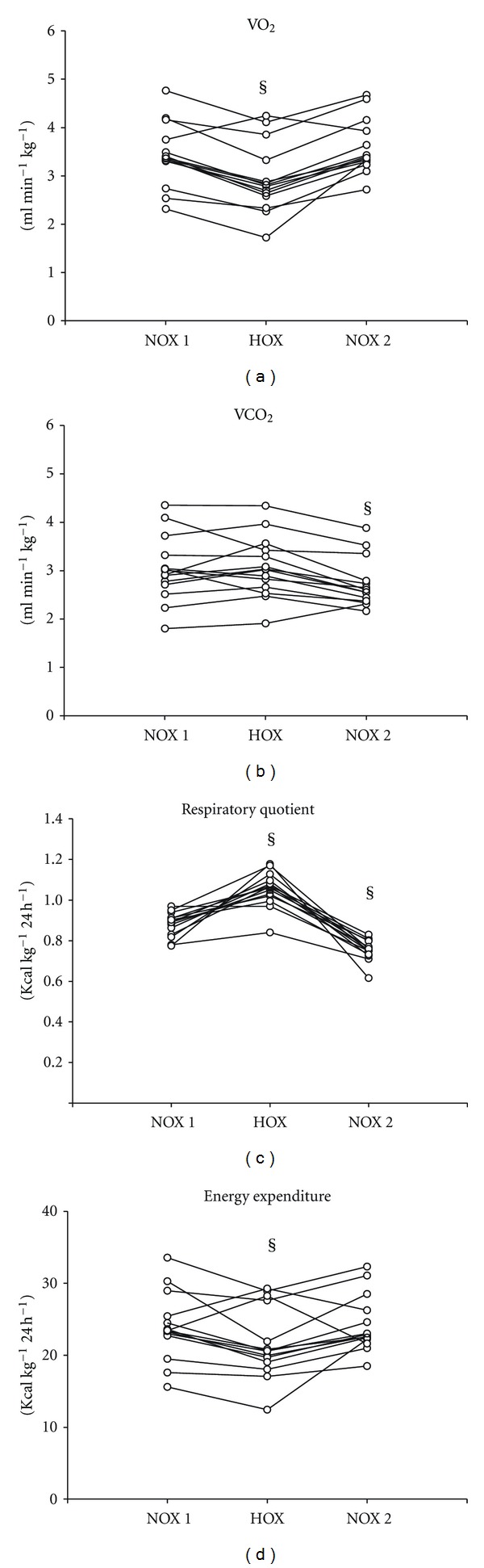
Single-experiment depiction of oxygen consumption (VO_2_), carbon dioxide production (VCO_2_), respiratory quotient (RR), and energy expenditure (EE) for the NOX 1 (baseline, FiO_2_ 0.21), HOX (FiO_2_ 1.0), and NOX 2 (FiO_2_ 0.21 again) time points. ^§^ *P* < 0.05 time point versus NOX 1.

Figure 1 and Table 2 illustrate the changes of VO_2_, VCO_2_, RQ, and EE during the 3 time points. After onset of HV, VO_2_ was reduced 18% at time point *HOX* (*P* < 0.05), whereas VCO_2_ remained unaltered (n.s.). Simultaneously, RQ increased by 22% (*P* < 0.05) and EE decreased by 12% (*P* < 0.05).  Table 1 depicts the hemodynamic changes during the study: HV slightly increased SaO_2_ and decreased HR (both *P* < 0.05). Arterial blood pressures (AOP_sys_, AOP_dia_) were not affected by HV. At time point *NOX 2*, VO_2_ returned to the value obtained before HV, whereas VCO_2_ and RQ were significantly decreased, even below the threshold of *NOX 1* (−13% and −27%, resp., both *P* < 0.05). EE returned to baseline at time point *NOX 2*.

## 4. Discussion

The main findings of this study were as follows. (1) Changes from normoxic to hyperoxic ventilation significantly reduced VO_2_. (2) After the onset of HV, the respiratory quotient (RQ) increased, whereas carbon dioxide production (VCO_2_) remained unaltered. (3) Most variables immediately returned to baseline when FiO_2_ was returned to 0.21 at time point *NOX 2*. Only VCO_2_ and RQ recovered slower and did not reach NOX 1 levels within the measurement period of NOX 2.

Whole-body VO_2_ can be measured by a pulmonary artery catheter or by a metabolic monitor. For resting patients breathing room air, both methods yielded satisfactory results for daily clinical practice. It is well known, however, that the accuracy of a standard metabolic monitor is rather low if FiO_2_ increases [19]. This is even more true for ventilation with pure oxygen. As a consequence, it has been impossible to use standard metabolic monitors to accurately determine VO_2_ above a maximum FiO_2_ of 0.6 due to technical problems (mainly the Haldane transformation; for technical details see Appendix). Therefore, all studies of hyperoxic ventilation and oxygen consumption have been conducted with a pulmonary artery catheter. However, this approach has several weaknesses, which cast the results obtained by this method into doubt [15–17, 20, 21]. Apart from the inferior reproducibility of the reversed Fick method, the most important finding is a consistent negative bias of calculated VO_2_ values versus calorimetric VO_2_ data observed by the majority of authors during normoxic conditions [22]. We determined VO_2,_ VCO_2_, and RQ during HV for the first time by using a modified metabolic monitor, which is not limited by these restrictions. We did not measure VO_2_ simultaneously by means of a pulmonary artery catheter during our protocol to directly compare the results of both methods. Since the main goal of our study was to determine VO_2_ during HV, placement of a pulmonary artery catheter might be judged an inappropriate risk for the subjects participating in the study. Furthermore, several studies already demonstrated a decline of VO_2_ during HV by means of a pulmonary artery catheter [10–14, 23, 24]. Using a modified metabolic monitor, we were able to replicate the results of these authors by a completely different technique. Consequently, we can state that HV actually decreases VO_2_, and that this phenomenon is unlikely to be judged a measurement artifact due to the method used. The fact that there are no studies validating the Delta Trac Pro for use with HV might be seen as a limitation to this study. Although the basic version of our metabolic monitor has been described and validated thoroughly elsewhere [18], there are no studies validating the Oxycon Pro during HV. This has to be stated as a limitation to our study.

Several mechanisms might be responsible for the observed decrease of VO_2_ during hyperoxic ventilation. It is well known that HV reduces heart rate and myocardial oxygen consumption [11, 25]. It might therefore be speculated that the HV-induced decline of whole-body VO_2_ might originate from a decrease of myocardial O_2_ consumption. However, because we observed only a negligible reduction of heart rate during HV, it seems unlikely that a concomitant decline of myocardial oxygen consumption is solely responsible for the observed decline of whole-body oxygen consumption.

Furthermore, it is known that breathing O_2_-enriched air transiently decreases minute ventilation by 10–20%, a phenomenon which might reduce respiratory work load and O_2_ consumption [26]. However, this effect, which has been attributed to a decrease in carotid body activity, lasts for less than 5 min [10]. Thereafter, minute ventilation returns to the baseline value and after another 5 min breathing of O_2_-enriched air increases minute ventilation up to 15% [27]. We therefore assume that this effect played a minor role in our setting.

Using a modified metabolic monitor for the determination of VO_2_ and VCO_2_ during HV yielded an additional result, which has not been observed previously: hyperoxic ventilation does not alter carbon dioxide production, despite a significant decline in oxygen consumption. This phenomenon might be explained by 2 different mechanisms: (1) During anaerobiosis, the VCO_2_/VO_2_ ratio (RQ) increases above 1.0, because alternative metabolic pathways (mainly anaerobic glycolysis) are engaged, using less-molecular oxygen for the production of the same amount of carbon dioxide. For example, the respiratory quotient increases during hypovolemia as soon as the anaerobic threshold is reached [28]. However, it seems very unlikely that HV resulted in severe anaerobic conditions in our setting, and this explanation might only play a minor role. (2) A second possible explanation for the decline of VO_2_ despite constant VCO_2_ during HV might be the fact that exposure to hyperoxia causes a substantial change in the metabolism of cells and tissues [29]. In Chinese hamster ovary cells exposed to hyperoxia for 24 h or more, Schoonen et al. found that the rate of oxygen consumption was substantially lower than that of cells maintained at normoxia [30]. The reduction in ATP generation from oxidative phosphorylation was partially offset by increased glycolysis; however, steady-state ATP levels were significantly reduced. One possible mechanism for this phenomenon is that aconitase, a mitochondrial matrix enzyme responsible for the hydration of citrate and isocitrate at the beginning of the citric acid cycle, is inactivated by exposure to hyperoxia [31]. These substantial changes in the oxidative pathway might at least partially explain the changes of VO_2_ despite constant VCO_2_ during HV. However, little is known about the different effects of HV on cellular oxygen metabolism in different organs *in vivo*, and therefore the relevance of this mechanism remains unclear. However, HV resulted in substantial changes of RQ in our model, and we speculate that changes of cellular O_2_ metabolism might, at least in part, be responsible for the changes of VO_2_ during HV.

The clinical impact of these HV-induced effects is ambiguous. HV is frequently used to treat hypoxemia and to preserve tissue oxygenation by increasing CaO_2_ during various pathological conditions where a critical restriction of oxygen transport is assumed (myocardial infarction, normovolemic anemia, hemorrhagic shock, etc.) [32–34]. However, it has been shown by many different investigators that HV regularly increases CaO_2_ but usually fails to increase local and systemic oxygen delivery (DO_2_) [11, 34–36]. This phenomenon is mainly attributed to the fact that HV induces generalized arteriolar constriction, which is accompanied by reduced functional capillary density [5–8] and nutritive organ blood flow, and increased peripheral oxygen shunting [8, 35]. As a consequence, HV-induced oxygen shunting might result in higher venous oxygen partial pressures and lower tissue oxygen partial pressures [7]. This should result in a reduction of VO_2_ at the expense of peripheral O_2_ delivery. However, we did not assess for signs of peripheral acidosis in our setting.

In summary, we speculate that the additional amount of O_2_ actually transported to the cells after initiation of HV might be negligible, since HV increases CaO_2_ but the accompanying decrease in nutritive organ blood flow prevents an increase of regional and whole-body DO_2_. Furthermore, this mechanism is in contrast to the beneficial effects of HV on oxygen transport and tissue oxygenation described above. One might speculate that the beneficial effects of HV during many different pathologies may to some extent be contributed to the fact that oxygen consumption of tissues is decreased by HV, and to the fact that HV increases CaO_2_. However, no clear proof of this concept is provided by the current data.

## 5. Conclusion

The change from normoxic to hyperoxic ventilation reduces whole-body oxygen consumption, regardless of the detection method, whereas carbon dioxide production (VCO_2_) remains unaltered. This phenomenon might be caused by substantial metabolic changes during HV; however, clarification of this phenomenon and its impact on oxygen transport and tissue oxygenation require further study.

## Figures and Tables

**Table 1:** Hemodynamic parameters.

	*NOX 1*	*HOX*	*NOX 2*
AOP_sys_ [mmHg]	115 (107/122)	115 (110/122)	118 (109/125)
AOP_dia_ [mmHg]	69 (66/74)	70 (66/78)	71 (67,10/76,90)
HR [min^−1^]	67 (60/77)	64 (56/71)^§^	66 (61/72)
SaO_2_ [%]	98 (97/98)	100 (99/100)^§^	98 (97/99)

Hemodynamic parameters. All values are presented as medians (Q1–Q3) for time points *NOX 1* (baseline, FiO_2_ 0.21), *HOX* (FiO_2_ 1.0), and *NOX 2* (FiO_2_ 0.21).

^§^ *P* < 0.05 time point versus *NOX 1.*

AOP_sys_: systolic arterial pressure; AOP_dia_: diastolic arterial pressure; HR: heart rate; SaO_2_: arterial hemoglobin saturation.

**Table 2:** Metabolic parameters.

	*NOX 1*	*HOX*	*NOX 2*
VO_2_ [mL/min/kg]	3.4 (3.0/4.0)	2.8 (2.5/3.6)^§^	3.4 (3.3/4.0)
RQ	0.9 (0.8/0.9)	1.1 (1.0/1.1)^§^	0.8 (0.7/0.8)^§^
VCO_2_ [mL/min/kg]	3.0 (2.6/3.6)	3.0 (2.6/3.5)	2.6 (2.4/3.1)^§^
EE [kcal kg^−1^ day^−1^]	23.4 (21.1/27.2)	20.6 (18.5/28.0)^§^	22.7 (21.9/27.4)

Metabolic parameters. All values are presented as medians (Q1–Q3) for time points *NOX 1* (baseline, FiO_2_ 0.21), *HOX* (FiO_2_ 1.0), and *NOX 2* (FiO_2_ 0.21).

^§^ *P* < 0.05 time point versus *NOX 1.*

VO_2_: oxygen consumption; RQ: respiratory quotient; VCO_2_: carbon dioxide production; EE: energy expenditure.

## Appendix

### The Haldane Transformation

Standard metabolic monitors quantify VO_2_, VCO_2_, and RQ by continuous measurement of the in- and expiratory oxygen fractions (F_*i*_O_2_ and F_*e*_O_2_). The difference F_*d*_O_2_ of F_*i*_O_2_ and F_*e*_O_2_ can be calculated as

(A.1) $$\begin{array}{c} {\text{F}}_{d}{\text{O}}_{2}={\text{F}}_{i}{\text{O}}_{2}-{\text{F}}_{e}{\text{O}}_{2\text{T}}. \end{array}$$

Using these values and the in- and expiratory CO_2_ concentrations (F_*i*_CO_2_ and F_*e*_CO_2_) and the Haldane transformation, the respiratory quotient can be calculated as follows:

(A.2) $$\begin{array}{c} \text{RQ}=\frac{1-{\text{F}}_{i}{\text{O}}_{2}}{\left({\text{F}}_{d}{\text{O}}_{2}/\left({\text{F}}_{e}\text{C}{\text{O}}_{2}-{\text{F}}_{i}\text{C}{\text{O}}_{2}\right)\right)-{\text{F}}_{i}{\text{O}}_{2}}. \end{array}$$

Because the respiratory quotient RQ is defined as

(A.3) $$\begin{array}{c} \text{RQ}=\frac{\text{VC}{\text{O}}_{2}}{\text{V}{\text{O}}_{2}}, \end{array}$$

VO_2_ can be calculated as

(A.4) $$\begin{array}{c} \text{V}{\text{O}}_{2}=\frac{\text{VC}{\text{O}}_{2}}{\text{RQ}}. \end{array}$$

However, using the Haldane transformation, an FiO_2_ of 1.0 will regularly result in a respiratory quotient of 0. Therefore, VO_2_ cannot be calculated this way for FiO_2_ = 1.0.

Calibration of the metabolic monitor and application of an adapted Haldane algorithm enables measurement of VO_2_ and VCO_2_ during hyperoxic conditions. However, the underlying algorithm has not been published by the manufacturer of the metabolic monitor.
